# Associations Between Sedentary Behaviors and Sedentary Patterns with Metabolic Syndrome in Children and Adolescents: The UP&DOWN Longitudinal Study

**DOI:** 10.3390/healthcare13192544

**Published:** 2025-10-09

**Authors:** Alejandro Sánchez-Delgado, Alejandro Perez-Bey, Julio Conde-Caveda, Rocío Izquierdo-Gómez, Sonia Gómez-Martínez, Oscar L. Veiga, Ascensión Marcos, José Castro-Piñero

**Affiliations:** 1 GALENO Research Group, Department of Physical Education, School of Education, University of Cádiz, 11519 Puerto Real, Spain; alejandro.sanchezdelgado@uca.es (A.S.-D.); julio.conde@uca.es (J.C.-C.); rocio.izquierdo@uca.es (R.I.-G.); jose.castro@uca.es (J.C.-P.); 2 Instituto de Investigación e Innovación Biomédica de Cádiz (INiBICA), 11009 Cádiz, Spain; 3 Immunonutrition Research Group, Department of Metabolism and Nutrition, Institute of Food Science and Technology and Nutrition (ICTAN)-CSIC, 28040 Madrid, Spain; songom03@ucm.es (S.G.-M.); amarcos@ictan.csic.es (A.M.); 4 EstiLIFE Research Group, Department of Physical Education, Sports and Human Movement, Autonomous University of Madrid, 28049 Madrid, Spain; oscar.veiga@uam.es

**Keywords:** cardiovascular disease, accelerometry, youth, sedentary bouts, sedentary lifestyle

## Abstract

Background/Objectives: The longitudinal associations between different modalities of sedentary behaviors (SBs) and sedentary patterns (SPs) with metabolic syndrome (MetS) in children and adolescents are unclear. We aimed to analyze the cross-sectional and longitudinal (2-year follow-up) association between SB and SP with the MetS score in Spanish children and adolescents. Methods: 76 children (34 females) and 186 adolescents (94 females) were included for SB analyses, and 175 children (82 females) and 188 adolescents (95 females) for SP. Children and adolescents were aged 6–11.9 years and 12–17.9 years, respectively. SB were assessed by a self-reported questionnaire and SP were determined by accelerometry. The MetS score was computed from the waist circumference, systolic blood pressure, triglycerides, high-density lipoprotein cholesterol, and glucose levels. Different linear regression models were implemented to examine cross-sectional, longitudinal, and change associations of SB and SP with MetS. Results: Total daily SB, educative daily SB, and mean SB were longitudinal and inversely associated with MetS (β = −0.001, all *p* < 0.05) in male adolescents, while other daily SB was longitudinal and inversely associated with MetS (β = −0.002, all *p* < 0.05) in female adolescents. Changes in screen and other daily SB were directly associated with MetS in female adolescents (β = 0.001 to 0.002, all *p* < 0.05). In contrast, changes in educative daily SB were inversely associated with MetS in female adolescents (β = −0.001, all *p* < 0.05). Conclusions: Few associations between SB modalities and the MetS score were found, mainly in adolescents and often in unexpected directions. In male adolescents, total and educative daily SB were negatively associated with MetS. In female adolescents, other daily SB and changes in educative daily SB showed negative associations, while changes in screen-based and other daily SB were positively associated with MetS. No associations were found between SP and MetS. Given the low evidence available to date, more longitudinal studies analyzing SB and SP simultaneously are needed to reach solid conclusions.

## 1. Introduction

Cardiovascular diseases (CVD) have persisted as the principal cause of mortality for more than three decades, representing nearly one-third of all deaths worldwide, with an estimated 20.5 million fatalities reported in 2021 [1]. Although CVD predominantly manifests after the fifth decade of life [2], its antecedents can already be identified during childhood [3]. Notably, CVD risk factors established in childhood and adolescence tend to persist into adulthood [4,5].

In this regard, the well-known metabolic syndrome (MetS) is defined as a cluster of risk factors for CVD and type 2 diabetes mellitus that occur together more often than by chance, strongly increasing the likelihood of developing CVD [6]. According to the International Diabetes Federation (IDF), MetS is defined by the presence of central obesity (waist circumference with ethnicity-specific values) plus any two of the following four factors: raised triglycerides (≥150 mg/dL or specific treatment), reduced high-density lipoprotein cholesterol (HDL-c; <40 mg/dL in men, <50 mg/dL in women or specific treatment), raised blood pressure (systolic ≥130 mmHg or diastolic ≥85 mmHg or treatment of previously diagnosed hypertension), and raised fasting plasma glucose (≥100 mg/dL or previously diagnosed type 2 diabetes) [7]. Recent evidence indicates that the prevalence of MetS is rising globally in tandem with increasing rates of obesity and sedentary behaviors, underlining its growing public health significance [8].

Several attempts have been made to define MetS in the pediatric population. However, the relatively low prevalence of CVD at these stages makes it difficult to define a discrete composite score of cardiovascular risk variables with sufficient capacity to predict future CVD [9]. Given this, various researchers have proposed the use of a continuous score that integrates multiple CVD risk factors into a single composite index, commonly referred to as the MetS score [10]. In this regard, a recent meta-analysis reported that the continuous MetS score represents a moderately accurate and cost-effective tool for predicting the risk of MetS in both young and adult populations [11]. Depending on the diagnostic criteria used, the prevalence of MetS cases among Spanish adolescents ranged from 5.5% to 14.9% in boys and from 3.4% to 32.6% in girls [12].

Sedentary behaviors (SB) have a fundamental role in the cardiovascular health of children and adolescents [13]. SB refers to any waking behavior characterized by an energy expenditure ≤ 1.5 metabolic equivalents, typically while in a sitting, reclining, or lying posture. Operationally, SB includes activities such as television viewing, computer use, or other screen-based entertainment performed while seated [14]. Beyond the total amount of sedentary time (ST), SB can also be characterized by modalities, which refer to the specific types of sedentary activities performed.

When analyzing SB modalities among the youth, most studies have focused on screen-based behaviors (i.e., TV viewing, video game and/or computer use). For instance, Carson et al. [15] reported that self-reported TV viewing and total screen time were unfavorably associated with body composition, clustered cardiometabolic risk, and behavioral conduct, with stronger evidence for television viewing than for other SB. Similarly, van Ekris et al. [16], concluded that childhood SB, especially screen time, was prospectively related to higher adiposity and unfavorable lipid and insulin profiles. Biddle et al. [17] further emphasized a likely causal relationship between sedentary screen behaviors and adiposity in youth, highlighting that the associations are more consistent and stronger for screen-based ST compared with total ST. In this line, SB, particularly screen-based activities, are highly prevalent among European youth. In the HELENA study, about one third of adolescents exceeded the recommended ≤2 h per day of screen time based solely on TV viewing during weekdays, and this prevalence increased to around 60% during weekends [18]. In Spain, findings from the ANIBES study confirmed this trend, showing that 48.4% of children and adolescents spent more than 2 h per day in front of a screen on average across the week, with 49.3% exceeding this threshold during weekdays and 84% during weekends [19]. However, screen time is only one SB modality, and there are a range of other modalities to consider, such as educative SB (i.e., reading or doing homework), social SB (i.e., listening to music or sitting and talking), or other SB (i.e., sitting doing nothing or motorized transport) [20]. Considering the full range of SB modalities provides a more complete picture of the lifestyle framework in relation to health-related variables [17]. Moreover, the majority of existing studies have explored the relationship between SB and MetS using cross-sectional designs [13], while evidence derived from longitudinal analyses remains limited.

Beyond ST and SB modalities, evidence from cross-sectional research indicates that sedentary patterns (SP), the manner in which ST accumulates (i.e., in blocks [bouts] of ≥10 min, ≥20 min, etc.), may play an important role in the associations with health measures, since shorter periods of ST appear to favor a healthier cardiometabolic profile [21,22]. In European children, ST tends to accumulate in short bouts (2–5 min) with fewer prolonged bouts (≥10 min), which are relatively uncommon [23]. To date, there is a limited number of longitudinal studies that examine ST using alternative grouping methods [24,25], and the duration of these studies is usually less than 2 years [26]. This lack of long-term evidence highlights the need for further research to clarify the role of ST and its patterns in youth cardiometabolic health. In addition, since previous studies have consistently reported sex- and age-related differences in SB and ST, stratifying analyses by these subgroups is warranted to provide a more accurate understanding of their associations with MetS [27,28,29].

The aims of the current study were (i) to analyze the cross-sectional and longitudinal associations between SB and SP with MetS risk in children and adolescents; (ii) to analyze associations between changes in SB and SP with MetS risk in children and adolescents.

## 2. Materials and Methods

### 2.1. Study Design and Population

The participants took part in the UP&DOWN study [30]. In brief, this 2-year longitudinal study aimed to assess the impact of physical activity (PA), SB, and health-related physical fitness on health indicators in Spanish youth. Schools were recruited through initial contact with headmasters and physical education teachers, followed by information sessions with parents and students. All students enrolled in 1st and 4th grades (6–7 and 9–10 years) in Cádiz and in 7th and 10th grades (12–13 and 15–16 years) in Madrid were invited to participate. Eligibility criteria included being apparently healthy, attending the participating schools, and having no physical disability or health condition that limited participation in PA.

The overall UP&DOWN cohort included 2264 participants (1226 children [580 girls] and 1038 adolescents [502 girls]). According to the Spanish Institute of National Statistics, the sample represents approximately 50% of schoolchildren in Cádiz and 5% of adolescents in Madrid. A random one-fourth of the cohort was invited to provide blood samples. The final baseline subsample with complete data on SB, body composition, systolic blood pressure (SBP), and blood biomarkers consisted of 104 children (45 girls) and 229 adolescents (110 girls). For SP, the corresponding baseline subsample comprised 229 children (106 girls) and 229 adolescents (110 girls).

Participants were followed for 2 years, with baseline data collected between September 2011 and June 2012 and follow-up data between September 2013 and June 2014. Only participants with complete baseline and follow-up data were included in the longitudinal analyses. After accounting for dropouts, the final analytical sample comprised 76 children (34 girls) and 186 adolescents (94 girls) for SB analyses (dropout rates of 26.9% and 18.8% in children and adolescents, respectively) and 175 children (82 girls) and 188 adolescents (95 girls) for SP analyses (dropout rates of 23.6% and 17.9%, respectively). Participants who withdrew from the study did so voluntarily. A descriptive comparison of baseline characteristics between participants who remained in the study and those who withdrew revealed no statistically significant differences in cardiometabolic, body composition, and PA variables. However, some differences were observed in sedentary behavior variables.

**Figure 1:**
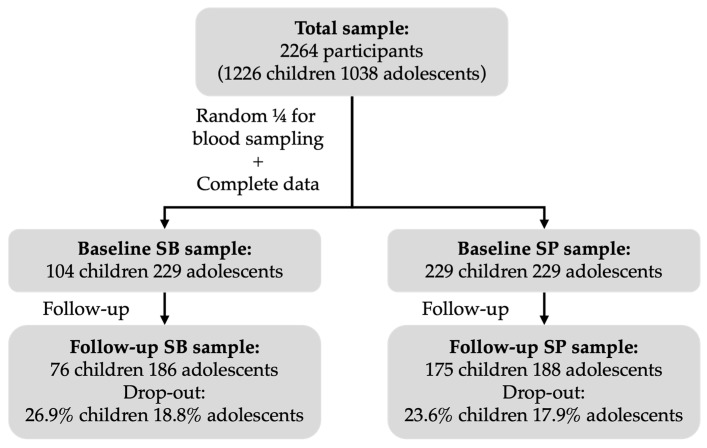
Flow diagram of participants through the UP&DOWN study for SB and SP analyses.

A diagrammatic representation of participant flow across study waves has been included (Figure 1). Written informed consent was provided by participants’ parents after being informed about the purposes of the study. The study protocol was accepted by the Ethics Committee of the Hospital Puerta del Hierro (Madrid, Spain), the Bioethics Committee of the National Spanish Research Council (Madrid, Spain) and the Committee for Research Involving Human Subjects of the University of Cádiz (Cádiz, Spain).

### 2.2. Tanner Stage

Explicative drawings of breast and genital development for females and males, respectively, were given to all participants for self-classification in one of the five stages of pubertal development, according to Tanner & Whitehouse [31].

### 2.3. Blood Pressure

SBP was measured with a validated digital automatic blood pressure monitor (Omron M6; Omron Healthcare Co., Ltd., Kyoto, Japan) according to the standardized and valid International Protocol of the European Society of Hypertension [32]. Two measurements were taken 1 to 2 min apart. If the first two readings differed in >5 mmHg, an additional measurement was taken, and the farthest value was removed. The average value of the two measurements was selected.

### 2.4. Blood Sampling

In the morning, after an overnight fast, 13.5 mL of blood was drawn through the cubital vein of each participant. Once the blood was collected, it was immediately transported to standard laboratories in each city. About 3.5 mL of the blood sample was collected in ethylenediaminetetraacetic acid (EDTA) and analyzed to acquire hemogram data. The remaining blood was collected in dried gel and sodium citrate and centrifuged to remove serum and plasma. Finally, serum and plasma were frozen at −80 °C for future analyses. In the current study, enzymatic colorimetric methods (Olympus AU2700 Analyzer; Olympus UK Ltd., Watford, UK) were used to analyze serum lipid triglycerides, HDL-c, and glucose.

### 2.5. Body Composition

Weight and height were measured with an electronic scale (Type SECA 861; range, 0.05–130 kg; precision, 0.05 kg; Hamburg, Germany) and a telescopic stature-measuring instrument (Type SECA 225; range, 60–200 cm; precision, 1 mm; Hamburg, Germany), respectively. These measurements were conducted with participants dressed in lightweight clothing and without shoes. Body mass index (BMI) was calculated as weight/height squared (kg/m^2^). Waist circumference (WC) was measured at the level of the narrowest part of the torso using a non-elastic tape (SECA 200; range, 0 to 150 cm; precision, 1 mm; Hamburg, Germany).

### 2.6. Metabolic Syndrome

MetS score was created from the mean of the standardized values of each individual CVD risk factor (i.e., WC, SBP, triglycerides, HDL-c, and glucose) by age group (children and adolescents) and sex (males and females). This index has been previously used by the International Diabetes Federation to assess cardiovascular health in children and adolescents [33]. The standardized value for HDL-c was multiplied by (−1) since higher HDL-c levels represent lower CVD risk.

### 2.7. Sedentary Behaviors

Self-reported SB were assessed using the Youth Leisure-time Sedentary Behavior Questionnaire (YLSBQ) [34]. This questionnaire assesses the amount of time (min/day) spent in 12 sedentary activities during the past week. Weekdays and weekend days were measured separately. The SB included (1) watching TV/videos, (2) playing computer/video games, (3) internet surfing, (4) doing homework/studying with a computer, (5) doing homework/studying without a computer, (6) sitting and talking, (7) sitting while doing nothing, (8) talking or chatting on the telephone (i.e., SMS or WhatsApp), (9) reading for fun, (10) listening to music, (11) doing cognitive hobbies such as jigsaw and crossword puzzles, chess, or checkers, and (12) traveling on motorized transport. Participants were instructed to report only the most relevant activity when two or more activities are performed at the same time.

Response options for items 1–7 were 0 min, 30 min, 1 h, 1 h and a half, 2 h, 2 h and a half, 3 h, 4 h, and 5 h or more. Response options for items 8–12 were 0 min, 15 min, 30 min, 1 h, 1 h and a half, 2 h, and 2 h and a half or more. The items were completed separately for weekdays and weekend days, referring to the last week. The average time per day spent on each behavior and each composite category was calculated as follows: [(weekday time × 5) + (weekend time × 2)]/7. Following the study of Cabanas-Sánchez et al. [34], we grouped SB into four categories by the sum (in minutes) of the following activities: screen time (SB items 1, 2, and 3, listed above), educational time (items 4, 5, and 9), social time (items 6, 8, and 10), and other time (items 7, 11, and 12). To minimize the overreporting phenomenon, an adjustment to leisure time was applied prior to the analysis and has been previously described in detail [34]. The adequacy of this scale was demonstrated by Cabanas-Sánchez et al. [34] by founding a good test–retest reliability (Intraclass Correlation Coefficient [ICC] = 0.76; Pearson’s correlation [r] = 0.78) and a moderate level of validity when comparing with accelerometry (r = 0.36).

### 2.8. Sedentary Time and Sedentary Patterns

ST was measured by ActiGraph accelerometer models GT1M, GT3X, and GT3X+ (Actigraph^TM^, LLC, Pensacola, FL, USA) at each time point. The GT1M accelerometers collected data at 2 s epochs, and the GT3X and GT3X+ collected data at 30 Hz. Devices were randomly assigned to participants at both baseline and follow-up, regardless of the model. Previous studies have demonstrated that there is strong agreement among outputs from the three Actigraph models [35,36]. The participants wore the accelerometer on the lower back, fitted with an elastic belt, during waking hours for 7 consecutive days and were instructed to remove them for time spent in bed and water-based activities [37]. Data were reintegrated into 10 s epochs before analysis [38]. Non-wearing time was defined as a period of 60 min of 0 counts and an allowance for up to two consecutives minutes of <100 counts per minute (CPM), with the up/down stream of 30 mins of consecutive 0 counts for a period for the detection of artifact movements [39]. Data were downloaded and analyzed by using the ACTILIFE software (v.6.11.7 Actigraph^TM^, Pensacola, FL, USA). Inclusion criteria for the analyses were (1) at least 3 days of valid data (2) and a minimum of 8h of registration per day [38]. Total ST (min/day) was calculated based on the recommended vector magnitude cut point [40]: <100 CPM. A bout was defined as any continuous period of ST and the bout stopped when the counts for an epoch went above the cut point for ST [41]. A break was defined as an interruption in ST and reflected any 5 s epoch change from ST to light-intensity PA or moderate to vigorous PA (MVPA). Total ST expressed in min/day was retained for analyses.

SP were characterized by the accumulation of ST in sustained, unbroken bouts of ≥10, ≥20, ≥30, or ≥45 min. For the present analyses, the following variables were included: (i) the number of bouts per day of 10 (N10), 20 (N20), 30 (N30), or 45 (N45) min; (ii) the total time accumulated per day in bouts of ≥10 (T10), ≥20 (T20), ≥30 (T30), or ≥45 (T45) min. We used the manufacturer’s software (v.6.11.7 Actigraph^TM^, Pensacola, FL, USA) for data download, reduction, cleaning, and analyses.

### 2.9. Moderate to Vigorous Physical Activity

MVPA was also registered by accelerometer. Epoch values that were equivalent to 2296 CPM were considered to be minutes of MVPA [40].

### 2.10. Data Analyses

Significant interactions by sex (males and females) and age group (children and adolescents) in the studied associations were observed. Consequently, all analyses were performed differentiating by sex and age groups. All variables were checked for normality through visual inspection using histograms and Q–Q plots. Descriptive statistics are presented as mean ± standard deviation. T-tests were used to analyze the differences in the variables of interest between sex for both age groups at both time points.

To examine the cross-sectional association of SB and SP with MetS score, linear regression models were used, where SB variables (i.e., total daily sedentary behavior (DSB), screen DSB, educative DSB, social DSB, other DSB, total weekend sedentary behavior (WSB), screen WSB, educative WSB, social WSB, other WSB, and mean SB) and SP variables (i.e., ST, N10, N20, N30, N45, T10, T20, T30, and T45) at baseline were individually introduced as independent variables and MetS scores at baseline were individually introduced as a dependent variable, all analyses were controlled for age, educational center, and mother’s education level at baseline (model 1). Model 2 was model 1 + MVPA at baseline.

To study the longitudinal association between SB and SP variables and MetS scores, linear regression models were used, where SB and SP variables at baseline were individually introduced as independent variables and MetS scores at 2-year follow-ups as a dependent variable. In model 1, we adjusted by age, educational center, mother’s education level, and MetS scores at baseline. Model 2 was model 1 + MVPA at baseline.

To analyze whether changes in SB and SP variables were associated with future MetS scores, linear regression models were used, where the changes (follow-up value—baseline value) in total DSB, screen DSB, educative DSB, social DSB, other DSB, total WSB, screen WSB, educative WSB, social WSB, other WSB, mean SB, ST, N10, N20, N30, N45, T10, T20, T30, and T45 were individually introduced as independent variables, and MetS scores at 2-year follow-ups were individually introduced as a dependent variable. In model 1, we adjusted by age, educational center, mother’s education level, and MetS score at baseline, and model 2 was model 1 + MVPA at baseline. The same analyses were performed but changes in MetS scores were introduced as a dependent variable in order to test whether changes in SB and SP variables were associated with changes in MetS. Analyses were controlled for the same variables excluding MetS at baseline. Analyses were performed using the environment for statistical computing R [42], version 4.0.3 (R Foundation for Statistical Computing, Vienna, Austria). The significance was set at *p* < 0.05.

## 3. Results

Participant characteristics are shown in Table 1. Overall, at baseline, female children and adolescents showed higher levels of HDL cholesterol (all *p* < 0.05) compared to male children and adolescents, respectively. Female adolescents had lower values of SBP, weight, height, and WC and higher values of SP (all *p* < 0.05) than male adolescents. Regarding SB, female adolescents had lower values of screen time and higher values of educative time compared to male adolescents (all *p* < 0.05). At the follow-up, female children had higher values of SP, except N10, (all *p* < 0.05) than male children. In adolescents, females presented a lower Tanner stage, SBP, weight, height, WC, MVPA, and higher HDL-c compared to male adolescents (all *p* < 0.05). Overall, female adolescents showed a higher SP than male adolescents (i.e., N10, N20, N30, and N45), all *p* < 0.05. In terms of SB, female adolescents had lower values of screen time and higher values of educative and social time compared to male adolescents (all *p* < 0.05).

Cross-sectional associations between SB and SP with MetS score at baseline are depicted in Table 2. Screen time in weekend days was negatively associated with MetS score in female children in model 1 and 2 (β = −0.001, all *p* < 0.05). N10 was positively associated with MetS score in female children in model 1 (β = 0.006, *p* = 0.029) but not in model 2 (β = 0.005, *p* = 0.053).

Table 3 shows the longitudinal associations between SB and SP variables at baseline and MetS scores at the 2-year follow-ups. Social weekend SB was negatively associated with the MetS score in male children only in model 1 (β = −0.002, *p* = 0.044). In male adolescents, the total daily SB, educative daily SB, and mean SB were negatively associated with the MetS score in models 1 and 2 (β = −0.001, all *p* < 0.05), while in female adolescents, other daily SB was negatively associated with the MetS score in both models (β = −0.002, all *p* < 0.05). No association was found between SP variables and the MetS score.

Supplementary Table S1 displays the changes in participant characteristics between the baseline and follow-up by age group and sex. Among children, females accumulated higher increases in sedentary bouts of ≥30 min compared to males (all *p* < 0.05). Among adolescents, females exhibited lower increases in SBP, weight, height, and WC but greater improvements in HDL cholesterol compared to males (all *p* < 0.05).

Table 4 shows the association between changes in SB and SP variables and the MetS score at follow-up. A slight decrease in screen and other daily SBs were positively associated with the MetS score in female adolescents in both models (β ranging from 0.001 to 0.002, all *p* < 0.05). In contrast, an increase in the educative daily SB was negatively associated with the MetS score in female adolescents in both models (β = −0.001, all *p* < 0.05). No association was found between SP variables and the MetS score.

The associations between changes in SB and SP variables with changes in the MetS score are depicted in Supplementary Table S2. A slight decrease in screen and other daily SBs were positively associated with the MetS score in female adolescents in both models (β ranging from 0.001 to 0.002, all *p* < 0.05). On the contrary, an increase in educative daily SB was negatively associated with the MetS score in female adolescents in both models (β = −0.001, all *p* < 0.05). No association was found between SP variables and the MetS score.

## 4. Discussion

The main results of our study indicate that the association between SB and MetS in the pediatric population is scarce and, in some cases, contrary to expectations. Overall, we found no significant relationship between SP and MetS risk. Only some associations were observed when differentiating by sex and SB modalities. Our results showed that screen WSB was cross-sectional and negatively associated with the MetS score in female children. Total DSB, educative DSB, and mean SB were longitudinal and negatively associated with the MetS score in male adolescents, while other DSB was longitudinal and negatively associated with the MetS score in female adolescents. Changes in screen and other DSBs were positively associated with the MetS score in female adolescents. In contrast, changes in educative DSB were negatively associated with the MetS score in female adolescents.

When analyzing SB modalities, a negative association was found between screen WSB and the MetS score in female children at the cross-sectional level, although a positive association between these two variables was expected [13,43,44,45]. This outcome could be partly explained by the fact that the female children group in our sample spent the lowest time in screen WSB. At the longitudinal level, negative associations between educative DSB, total DSB, and the mean of SB with the MetS score were found in male adolescents, while in female adolescents, a negative association was shown between other DSB with the MetS score. When changes in SBs were analyzed, we found positive associations between a slight decrease in screen DSB and other DSB with the MetS score. In the literature, a longer screen time duration has been associated with higher MetS risk [13]. This result could be explained by the fact that longer screen time is closely associated with a worsening of dietary habits and lifestyle profiles among young people [46]. In contrast, negative longitudinal associations were observed between changes in educational DSB and the MetS score in adolescent females, suggesting that a reduction in educational DSB may be linked to an increase in cardiometabolic risk. In this regard, Stephens et al. [47] analyzed the role of education across a broad spectrum of educational levels (from primary school to doctoral degree), showing that higher education correlated with significantly better metabolic health compared to lower levels. This finding could indicate that the modality of SBs could affect the cardiovascular health of children and adolescents.

Our results did not show any associations between the total ST and MetS. Our findings contrast with traditional evidence linking sedentary lifestyles to poorer metabolic health in young people. Multiple cross-sectional studies have reported that greater exposure to SB (with total ST representing the overall duration in minutes) is associated with an increased risk of obesity and MetS from an early age [15,48]. A recent national study in China, with more than 17,000 participants aged 7 to 17, confirmed that children and adolescents with high levels of SB had a higher prevalence of abdominal obesity, dyslipidemia, and a significantly elevated risk of MetS [48]. Consistently, a Brazilian multicenter study reported that adolescents who spent more than 8 h a day sitting were twice as likely to have MetS compared to their less sedentary peers [49]. In that cohort, ST was positively related to central adiposity and an adverse lipid profile, while MVPA showed a positive association with improved insulin sensitivity [49]. This evidence reinforces the notion that prolonged ST contributes to an unfavorable metabolic profile in young people.

However, the literature also shows heterogeneous results, partly in line with our observations. A previous meta-analysis [50] found no independent association between total ST and MetS risk once MVPA levels were controlled for. This suggests that the apparent impact of ST may be mediated or confined to contexts of low PA. In active populations, sitting for long periods may not have such a marked effect on metabolic risk. In fact, in our study, the introduction of MVPA as a covariate eliminated the only initial association observed between sedentary bouts of ≥10 min and MetS. For its part, a recent longitudinal study also found that screen time, sleep, or PA did not have significant cross-sectional effects on cardiometabolic risk scores in children and adolescents [51]. In that study, the main predictor of future MetS risk was the participants’ baseline adiposity status. This may suggest that the impact of ST on metabolic health manifests primarily in the context of excess body weight, in line with previous findings [52].

Regarding SP, the way in which ST accumulates, our results are consistent with previous studies that also found no clear associations in young people [53]. One possible explanation is that most children and adolescents spontaneously interrupt their sedentary periods very frequently (i.e., they rarely remain seated for ≥30–60 continuous minutes) [54], so they do not reach thresholds of prolonged SB sufficient to trigger measurable metabolic effects. In line with this, our study observed only a limited number of bouts lasting ≥30 min, further reinforcing the notion that prolonged periods of SB are uncommon in this population. Additionally, the lack of significant associations with SP could also be partially explained by limited statistical power within certain subgroups, especially when stratifying by age and sex. The relatively low frequency of prolonged sedentary bouts may have reduced the variability needed to detect meaningful relationships, increasing the risk of type II error. Therefore, null findings should be interpreted with caution.

Overall, we found no evidence that SB modalities and total ST or SP are directly related to MetS risk in children and adolescents in our study. The observed associations were scarce, restricted to certain subgroups, and often contrary to the initially hypothesized direction. This suggests that the influence of SB on youth metabolic health may be more complex than anticipated and, to some extent, dependent on other variables, such as PA or adiposity, as suggested by our results and complemented by the previous literature.

### Limitations and Strengths

Several limitations should be mentioned. First, given the observational nature of the study, causal inferences cannot be established. Second, sample loss may have introduced a potential selection bias, as participants who dropped out differed from those who remained in some indicators of sedentary behavior (i.e., greater daily and social ST and less weekly ST). This may limit the representativeness of the final sample and should be considered when interpreting the results. Third, the generalization of these results should be considered cautiously because we could not determine the influence of ethnicity and a country’s economic development on these associations, given that only urban and Caucasian Spanish youths participated in this study.

Furthermore, we acknowledge that multiple comparisons were conducted when evaluating different modalities and patterns of sedentary behavior. Although the models were adjusted for potential confounders (age, educational center, mother’s education level, and, additionally, MVPA time), these adjustments do not address the issue of multiplicity. Given the primarily exploratory and hypothesis-generating nature of the study, we chose not to apply formal corrections (i.e., Bonferroni/FDR) to avoid increasing the risk of type II errors and overlooking potentially relevant associations in pediatric populations. Therefore, the findings, particularly those with *p*-values close to the significance threshold, should be interpreted with caution, emphasizing the magnitude and consistency of the effects rather than isolated statistical significance, and require independent confirmation in external cohorts.

Finally, this study focused on SB modalities during leisure time and did not capture time spent in SB during school hours (i.e., screen time during recess) or whether two or more SBs were occurring simultaneously. Youth may engage in multiple SBs across multiple social media devices at the same time (i.e., smartphone use while watching TV); future studies could therefore include measures that capture multiple SBs simultaneously. Otherwise, the current research presents some strengths. The longitudinal design and the relatively large sample, which allowed us to conduct the analyses differentiating by sex and age groups, are major strengths of the present study. This stratification is particularly relevant given that previous research has consistently reported differences in SB and SP according to sex and age. For instance, females generally present a higher ST than males [27,28], while males tend to spend more time on screen-based activities [29]. In addition, ST seems to increase with age, with adolescents presenting higher ST levels [28]. Apart from this, the use of clustered MetS risk factors has been suggested as a good indicator of cardiovascular health compared with individual MetS risk factors [5]. Finally, we simultaneously analyzed SB and SP modalities, while most studies only analyze SB (focusing on screen time) [55] or SP (focusing on a single-bout duration, i.e., bouts > 10 min or bouts > 30 min of ST).

## 5. Conclusions

In conclusion, few cross-sectional and longitudinal associations between SB or SP and the MetS score were observed. These associations were mainly detected in adolescents and often in directions contrary to the initial hypotheses. Regarding changes over the two-year follow-up, positive associations with the MetS score were found for increases in screen-based and other daily SBs, whereas negative associations were observed for changes in educational SB, all restricted to female adolescents. No associations were detected for total ST or SP. Taken together, these findings highlight the need for future studies to clarify the mechanisms through which SB influences cardiometabolic health and to determine whether these effects depend on other features, such as PA or adiposity levels.

## Figures and Tables

**Table 1:** Baseline and 2 y follow-up characteristics of the study sample by age and sex.

Baseline						
	Male children	Female children	*p* value	Male adolescents	Female adolescents	*p* value
Sedentary behaviors	(*n* = 59)	(*n* = 45)		(*n* = 119)	(*n* = 110)	
Total DSB (min/day)	306.77 (116.29)	312.31 (132.20)	0.821	416.85 (127.19)	433.27 (127.73)	0.331
Screen DSB (min/day)	101.63 (96.29)	80.42 (91.69)	0.259	156.95 (124.42)	113.35 (106.36)	**0.005**
Educative DSB (min/day)	142.24 (105.23)	147.87 (110.85)	0.792	162.18 (125.30)	215.49 (135.18)	**0.002**
Social DSB (min/day)	43.18 (42.43)	66.99 (75.37)	**0.044**	65.14 (94.12)	76.78 (78.54)	0.313
Other DSB (min/day)	19.72 (27.91)	17.03 (25.86)	0.616	32.59 (49.68)	27.64 (51.22)	0.459
Total WSB (min/day)	516.27 (158.62)	484.79 (174.32)	0.339	571.50 (188.12)	577.85 (161.95)	0.785
Screen WSB (min/day)	282.36 (160.44)	228.97 (167.72)	0.102	312.00 (180.89)	238.66 (141.36)	**0.001**
Educative WSB (min/day)	93.25 (82.34)	99.06 (83.01)	0.723	113.90 (101.18)	150.62 (121.97)	**0.014**
Social WSB (min/day)	90.68 (70.39)	109.24 (82.30)	0.219	93.90 (85.59)	137.73 (115.19)	**0.001**
Other WSB (min/day)	49.97 (57.54)	47.52 (47.09)	0.817	51.71 (75.29)	50.85 (61.75)	0.925
Mean SB (min/day)	366.63 (101.57)	361.59 (129.40)	0.824	461.04 (119.71)	474.58 (109.36)	0.374
Sedentary pattern	(*n* = 123)	(*n* = 106)		(*n* = 119)	(*n* = 110)	
Accelerometer wear time (h/day)	13.65 (0.8)	13.5 (0.74)	0.398	14.27 (1.81)	14.26 (1.52)	0.947
Sedentary time (min/day)	520.60 (69.04)	537.72 (72.85)	0.069	650.22 (116.81)	686.07 (100.40)	**0.014**
Bouts of 10 min (number/day)	5.98 (3.14)	6.71 (3.15)	0.079	12.03 (5.06)	14.26 (4.56)	**0.001**
Time in bouts of 10 min (min/day)	104.91 (60.78)	117.85 (59.06)	0.105	218.97 (113.25)	279.41 (110.48)	**<0.001**
Bouts of 20 min (number/day)	1.39 (1.08)	1.61 (1.03)	0.12	3.21 (2.32)	4.75 (2.42)	**<0.001**
Time in bouts of 20 min (min/day)	43.80 (35.38)	49.73 (33.84)	0.198	99.48 (79.16)	148.68 (82.86)	**<0.001**
Bouts of 30 min (number/day)	0.54 (0.54)	0.58 (0.49)	0.537	1.26 (1.18)	1.98 (1.31)	**<0.001**
Time in bouts of 30 min (min/day)	23.30 (23.96)	25.00 (22.71)	0.584	52.46 (52.39)	81.83 (57.30)	**<0.001**
Bouts of 45 min (number/day)	0.18 (0.25)	0.19 (0.22)	0.669	0.38 (0.51)	0.59 (0.56)	**0.003**
Time in bouts of 45 min (min/day)	10.35 (15.02)	11.15 (13.95)	0.678	20.68 (28.89)	31.58 (31.10)	**0.006**
Physical activity						
MVPA (min/day)	69.00 (21.06)	52.73 (18.61)	**<0.001**	61.11 (19.31)	44.68 (16.83)	**<0.001**
Tanner stage	1.59 (0.63)	1.45 (0.72)	0.137	3.61 (0.92)	3.42 (0.75)	0.081
Age (years)	8.09 (1.52)	8.05 (1.54)	0.833	14.10 (1.64)	13.87 (1.47)	0.266
Systolic blood pressure (mmHg)	101.82 (10.74)	99.25 (11.32)	0.081	111.16 (13.84)	106.27 (9.88)	**0.003**
Triglycerides (mg/dL)	40.10 (18.75)	45.67 (17.91)	**0.023**	48.12 (19.17)	53.99 (22.16)	**0.033**
HDL cholesterol (mg/dL)	39.98 (16.30)	41.63 (15.86)	0.44	48.14 (15.34)	49.55 (14.70)	0.481
Glucose (mg/dL)	61.42 (17.86)	62.83 (16.53)	0.539	79.55 (15.90)	77.58 (15.63)	0.345
Body composition						
Weight (kg)	30.54 (8.12)	31.16 (11.07)	0.626	55.22 (12.92)	52.19 (9.39)	**0.044**
Height (cm)	128.78 (9.96)	129.23 (12.11)	0.753	162.86 (12.23)	157.85 (6.62)	**<0.001**
Body mass index (kg/m^2^)	18.14 (2.76)	18.14 (3.63)	0.996	20.57 (2.93)	20.86 (3.01)	0.462
Waist circumference (cm)	59.32 (6.81)	58.15 (8.59)	0.255	69.19 (7.05)	66.06 (5.79)	**<0.001**
Follow-up						
	Male children	Female children	*p* value	Male adolescents	Female adolescents	*p* value
Sedentary behaviors	(*n* = 42)	(*n* = 34)		(*n* = 92)	(*n* = 94)	
Total DSB (min/day)	323.71 (131.78)	276.82 (162.85)	0.169	444.57 (133.05)	481.87 (116.84)	**0.044**
Screen DSB (min/day)	111.15 (114.07)	83.18 (81.49)	0.233	145.68 (132.10)	102.24 (107.48)	**0.015**
Educative DSB (min/day)	138.22 (110.18)	136.29 (119.87)	0.942	191.15 (134.53)	256.79 (172.84)	**0.004**
Social DSB (min/day)	44.50 (46.22)	44.12 (49.70)	0.973	79.97 (71.97)	103.28 (78.10)	**0.036**
Other DSB (min/day)	29.84 (58.60)	13.23 (18.09)	0.116	27.78 (35.62)	19.56 (26.82)	0.077
Total WSB (min/day)	554.61 (186.73)	489.59 (232.67)	0.181	617.31 (183.46)	616.07 (161.73)	0.961
Screen WSB (min/day)	314.51 (167.32)	223.64 (195.26)	**0.032**	297.34 (192.70)	180.02 (137.74)	**<0.001**
Educative WSB (min/day)	109.66 (109.90)	105.19 (84.50)	0.846	159.34 (174.67)	230.53 (197.24)	**0.010**
Social WSB (min/day)	86.58 (98.48)	117.20 (128.39)	0.243	120.20 (94.83)	174.64 (114.85)	**0.001**
Other WSB (min/day)	43.85 (55.77)	43.57 (62.93)	0.984	40.43 (44.56)	30.88 (30.85)	0.090
Mean SB (min/day)	389.68 (127.86)	337.62 (159.77)	0.119	493.93 (120.87)	520.21 (108.93)	0.121
Sedentary pattern	(*n* = 93)	(*n* = 82)		(*n* = 93)	(*n* = 95)	
Accelerometer wear time (h/day)	10.17 (1.78)	10.35 (1.74)	0.654	10.62 (2.7)	11.51 (2.12)	**0.013**
Sedentary time (min/day)	558.70 (64.50)	577.73 (65.53)	0.055	849.72 (426.81)	877.48 (370.85)	0.634
Bouts of 10 min (number/day)	8.07 (3.59)	8.93 (3.45)	0.11	14.60 (5.44)	16.81 (4.70)	**0.003**
Time in bouts of 10 min (min/day)	136.29 (70.88)	161.38 (75.27)	**0.024**	423.71 (392.71)	489.49 (339.19)	0.22
Bouts of 20 min (number/day)	1.75 (1.39)	2.36 (1.58)	**0.007**	4.63 (2.63)	6.39 (2.79)	**<0.001**
Time in bouts of 20 min (min/day)	51.66 (43.02)	72.12 (53.19)	**0.006**	288.14 (380.88)	345.62 (329.10)	0.269
Bouts of 30 min (number/day)	0.58 (0.60)	0.83 (0.80)	**0.017**	1.98 (1.54)	3.08 (1.81)	**<0.001**
Time in bouts of 30 min (min/day)	23.91 (25.77)	35.19 (34.69)	**0.015**	224.55 (371.97)	265.80 (324.72)	0.419
Bouts of 45 min (number/day)	0.16 (0.23)	0.27 (0.35)	**0.009**	0.80 (0.91)	1.25 (1.16)	**0.004**
Time in bouts of 45 min (min/day)	8.91 (13.62)	15.26 (19.22)	**0.012**	181.91 (365.67)	199.57 (316.13)	0.723
Physical activity						
MVPA (min/day)	60.04 (18.77)	53.30 (22.60)	**0.032**	56.30 (27.37)	42.15 (19.75)	**<0.001**
Tanner stage	2.31 (0.59)	2.16 (1.06)	0.232	4.45 (0.62)	3.98 (0.62)	**<0.001**
Age (years)	10.14 (1.50)	10.18 (1.50)	0.865	16.00 (1.60)	15.81 (1.43)	0.393
Systolic blood pressure (mmHg)	105.92 (9.04)	105.42 (10.55)	0.734	113.57 (12.39)	103.67 (9.49)	**<0.001**
Triglycerides (mg/dL)	40.73 (19.31)	45.32 (24.66)	0.17	63.53 (25.39)	62.17 (22.98)	0.701
HDL cholesterol (mg/dL)	39.95 (14.97)	40.33 (16.52)	0.872	52.02 (12.28)	59.58 (11.48)	**<0.001**
Glucose (mg/dL)	71.57 (16.91)	73.21 (17.08)	0.526	86.94 (7.94)	84.71 (8.07)	0.058
Body composition						
Weight (kg)	38.56 (10.23)	40.18 (13.28)	0.365	62.66 (10.88)	56.26 (8.88)	**<0.001**
Height (cm)	140.69 (10.07)	143.31 (12.29)	0.123	171.83 (7.83)	162.18 (5.17)	**<0.001**
Body mass index (kg/m^2^)	19.21 (3.32)	19.10 (4.05)	0.844	21.14 (2.98)	21.40 (3.24)	0.58
Waist circumference (cm)	63.37 (8.41)	61.24 (9.23)	0.112	71.80 (6.75)	67.15 (6.61)	**<0.001**

DSB, daily sedentary behaviors; HDL, high density lipoprotein; MVPA, moderate to vigorous physical activity; SB, sedentary behaviors; and WSB, weekend sedentary behaviors. Values are presented as mean (standard deviation). Statistically significant differences between sex in variables are highlighted in bold.

**Table 2:** Cross-sectional association between sedentary behaviors and sedentary patterns with metabolic syndrome score in children and adolescents.

Sedentary behaviors												
	Children (*n* = 104)						Adolescents (*n* = 229)					
	Male (*n* = 59)			Female (*n* = 45)			Male (*n* = 119)			Female (*n* = 110)		
	Adjusted R^2^	β	*p*	Adjusted R^2^	β	*p*	Adjusted R^2^	β	*p*	Adjusted R^2^	β	*p*
Model 1												
Total DSB	−0.023	0.001	0.175	0.292	0.000	0.824	0.264	0.001	0.134	0.039	0.000	0.303
Screen DSB	−0.021	0.001	0.167	0.320	−0.001	0.283	0.249	0.000	0.584	0.036	0.000	0.385
Educative DSB	−0.070	0.000	0.924	0.309	−0.001	0.403	0.253	0.000	0.362	0.028	0.000	0.885
Social DSB	−0.070	0.000	0.897	0.362	0.002	0.090	0.267	0.001	0.104	0.032	0.000	0.523
Other DSB	−0.070	0.000	0.850	0.301	0.003	0.537	0.261	0.001	0.173	0.029	0.000	0.750
Total WSB	−0.070	0.000	0.868	0.334	−0.001	0.190	0.259	0.000	0.215	0.035	0.000	0.430
Screen WSB	−0.069	0.000	0.805	0.389	−0.001	**0.044**	0.260	0.000	0.189	0.033	0.000	0.491
Educative WSB	−0.029	0.001	0.206	0.292	0.000	0.899	0.253	0.000	0.393	0.054	0.001	0.118
Social WSB	−0.010	−0.002	0.124	0.307	0.001	0.426	0.247	0.000	0.933	0.040	0.000	0.279
Other WSB	−0.037	0.001	0.255	0.300	0.001	0.551	0.257	0.001	0.260	0.031	0.000	0.611
Mean SB	−0.035	0.001	0.240	0.304	0.000	0.484	0.270	0.001	0.081	0.043	0.001	0.230
Model 2												
Total DSB	0.056	0.001	0.185	0.269	0.000	0.837	0.277	0.000	0.238	0.047	0.000	0.407
Screen DSB	0.069	0.001	0.130	0.297	−0.001	0.305	0.269	0.000	0.616	0.052	0.001	0.274
Educative DSB	0.014	0.000	0.833	0.289	−0.001	0.388	0.273	0.000	0.367	0.042	0.000	0.655
Social DSB	0.015	0.001	0.773	0.342	0.002	0.093	0.284	0.001	0.133	0.043	0.000	0.564
Other DSB	0.014	0.000	0.863	0.282	0.004	0.476	0.274	0.001	0.349	0.041	0.000	0.751
Total WSB	0.014	0.000	0.818	0.315	−0.001	0.184	0.278	0.000	0.236	0.044	0.000	0.511
Screen WSB	0.013	0.000	0.934	0.368	−0.001	**0.049**	0.279	0.000	0.202	0.045	0.000	0.477
Educative WSB	0.053	0.001	0.202	0.269	0.000	0.867	0.269	0.000	0.661	0.058	0.000	0.188
Social WSB	0.058	−0.001	0.174	0.286	0.001	0.419	0.267	0.000	0.834	0.052	0.000	0.287
Other WSB	0.024	0.001	0.508	0.275	0.001	0.629	0.272	0.001	0.395	0.041	0.000	0.683
Mean SB	0.047	0.001	0.239	0.281	0.000	0.489	0.283	0.001	0.139	0.050	0.000	0.327
Sedentary patterns												
	Children (*n* = 229)						Adolescents (*n* = 229)					
	Male (*n* = 123)			Female (*n* = 106)			Male (*n* = 119)			Female (*n* = 110)		
	Adjusted R^2^	β	*p*	Adjusted R^2^	β	*p*	Adjusted R^2^	β	*p*	Adjusted R^2^	β	*p*
Model 1												
Sedentary time	0.099	0.000	0.726	0.154	0.000	0.157	0.249	0.000	0.611	0.036	0.000	0.375
Bouts of 10 min	0.120	0.003	0.114	0.181	0.006	**0.029**	0.248	0.000	0.764	0.056	0.002	0.103
Time in bouts of 10 min	0.113	0.000	0.187	0.169	0.000	0.060	0.250	0.000	0.523	0.050	0.000	0.146
Bouts of 20 min	0.114	0.008	0.175	0.147	0.009	0.262	0.250	−0.002	0.547	0.041	0.003	0.277
Time in bouts of 20 min	0.108	0.000	0.291	0.146	0.000	0.291	0.253	0.000	0.382	0.041	0.000	0.277
Bouts of 30 min	0.111	0.014	0.228	0.136	0.006	0.700	0.259	−0.008	0.208	0.046	0.007	0.192
Time in bouts of 30 min	0.105	0.000	0.401	0.139	0.000	0.517	0.261	0.000	0.167	0.044	0.000	0.216
Bouts of 45 min	0.099	−0.007	0.759	0.141	0.027	0.434	0.269	−0.024	0.089	0.034	0.009	0.452
Time in bouts of 45 min	0.099	0.000	0.747	0.144	0.001	0.319	0.270	0.000	0.082	0.034	0.000	0.451
Model 2												
Sedentary time	0.112	0.000	0.851	0.158	0.000	0.180	0.269	0.000	0.594	0.052	0.000	0.272
Bouts of 10 min	0.127	0.003	0.179	0.176	0.005	0.053	0.270	−0.001	0.561	0.071	0.002	0.084
Time in bouts of 10 min	0.123	0.000	0.253	0.166	0.000	0.105	0.273	0.000	0.375	0.067	0.000	0.105
Bouts of 20 min	0.125	0.007	0.210	0.147	0.008	0.380	0.272	−0.002	0.414	0.057	0.004	0.206
Time in bouts of 20 min	0.120	0.000	0.319	0.146	0.000	0.410	0.275	0.000	0.288	0.058	0.000	0.190
Bouts of 30 min	0.122	0.012	0.269	0.140	0.003	0.841	0.281	−0.008	0.165	0.067	0.008	0.109
Time in bouts of 30 min	0.117	0.000	0.420	0.142	0.000	0.644	0.284	0.000	0.134	0.065	0.000	0.122
Bouts of 45 min	0.112	−0.003	0.891	0.144	0.022	0.524	0.289	−0.024	0.087	0.051	0.013	0.300
Time in bouts of 45 min	0.112	0.000	0.874	0.147	0.000	0.399	0.290	0.000	0.077	0.052	0.000	0.287

β, standardized coefficient; DSB, daily sedentary behaviors; SB, sedentary behaviors; and WSB, weekend sedentary behaviors. Model 1: Analyses were controlled by age, educational center, and mother’s education level. Model 2: Model 1 plus moderate to vigorous physical activity time. Statistically significant values are highlighted in bold.

**Table 3:** Longitudinal association between sedentary behavior and sedentary patterns with metabolic syndrome score in children and adolescents.

Sedentary behaviors												
	Children (*n* = 76)						Adolescents (*n* = 186)					
	Male (*n* = 42)			Female (*n* = 34)			Male (*n* = 92)			Female (*n* = 94)		
	Adjusted R^2^	β	*p*	Adjusted R^2^	β	*p*	Adjusted R^2^	β	*p*	Adjusted R^2^	β	*p*
Model 1												
Total DSB	0.275	0.000	0.760	0.219	0.000	0.686	0.587	−0.001	**0.006**	0.442	0.000	0.593
Screen DSB	0.273	0.000	0.849	0.211	0.000	0.936	0.542	0.000	0.980	0.443	0.000	0.502
Educative DSB	0.277	0.000	0.678	0.293	−0.001	0.179	0.581	−0.001	**0.011**	0.456	0.001	0.141
Social DSB	0.296	−0.002	0.372	0.336	0.003	0.091	0.546	−0.001	0.393	0.441	0.000	0.632
Other DSB	0.288	−0.002	0.473	0.220	−0.003	0.655	0.550	0.001	0.261	0.471	−0.002	**0.040**
Total WSB	0.278	0.000	0.654	0.225	0.000	0.584	0.542	0.000	0.982	0.444	0.000	0.425
Screen WSB	0.356	0.001	0.090	0.224	0.000	0.598	0.545	0.000	0.456	0.440	0.000	0.862
Educative WSB	0.304	−0.001	0.300	0.212	0.000	0.910	0.561	−0.001	0.081	0.441	0.000	0.629
Social WSB	0.387	−0.002	**0.044**	0.239	0.001	0.439	0.542	0.000	0.783	0.443	0.000	0.494
Other WSB	0.273	0.000	0.867	0.271	−0.002	0.251	0.549	0.001	0.288	0.440	0.000	0.846
Mean SB	0.272	0.000	0.973	0.211	0.000	0.966	0.568	−0.001	**0.038**	0.444	0.000	0.426
Model 2												
Total DSB	0.250	0.000	0.692	0.170	0.000	0.695	0.592	−0.001	**0.004**	0.439	0.000	0.668
Screen DSB	0.247	0.000	0.777	0.162	0.000	0.936	0.542	0.000	0.976	0.440	0.000	0.585
Educative DSB	0.251	0.000	0.670	0.250	−0.001	0.188	0.582	−0.001	**0.011**	0.451	0.000	0.184
Social DSB	0.274	−0.002	0.341	0.295	0.003	0.101	0.548	−0.001	0.358	0.439	0.000	0.666
Other DSB	0.260	−0.002	0.496	0.172	−0.003	0.662	0.547	0.001	0.376	0.468	−0.002	**0.042**
Total WSB	0.251	0.000	0.665	0.177	0.000	0.588	0.542	0.000	0.960	0.441	0.000	0.476
Screen WSB	0.330	0.001	0.100	0.176	0.000	0.608	0.546	0.000	0.451	0.438	0.000	0.866
Educative WSB	0.285	−0.001	0.266	0.162	0.000	0.909	0.557	−0.001	0.129	0.438	0.000	0.723
Social WSB	0.363	−0.002	0.050	0.192	0.001	0.451	0.544	0.000	0.681	0.441	0.000	0.515
Other WSB	0.245	0.000	0.951	0.242	−0.003	0.210	0.548	0.001	0.332	0.438	0.000	0.781
Mean SB	0.245	0.000	0.918	0.162	0.000	0.968	0.571	−0.001	**0.034**	0.441	0.000	0.502
Sedentary patterns												
	Children (*n* = 175)						Adolescents (*n* = 188)					
	Male (*n* = 93)			Female (*n* = 82)			Male (*n* = 93)			Female (*n* = 95)		
	Adjusted R^2^	β	*p*	Adjusted R^2^	β	*p*	Adjusted R^2^	β	*p*	Adjusted R^2^	β	*p*
Model 1												
Sedentary time	0.472	0.000	0.472	0.527	0.000	0.405	0.537	0.000	0.785	0.457	0.000	0.482
Bouts of 10 min	0.469	0.001	0.677	0.522	0.000	0.845	0.536	0.000	0.814	0.453	0.000	0.992
Time in bouts of 10 min	0.468	0.000	0.855	0.523	0.000	0.634	0.536	0.000	0.967	0.453	0.000	0.826
Bouts of 20 min	0.468	−0.001	0.822	0.524	0.005	0.552	0.536	0.000	0.986	0.453	0.000	0.930
Time in bouts of 20 min	0.468	0.000	0.804	0.527	0.000	0.383	0.536	0.000	0.909	0.454	0.000	0.764
Bouts of 30 min	0.468	0.001	0.915	0.528	0.016	0.358	0.536	0.000	0.956	0.454	0.001	0.813
Time in bouts of 30 min	0.468	0.000	0.928	0.532	0.000	0.239	0.536	0.000	0.936	0.455	0.000	0.617
Bouts of 45 min	0.468	−0.003	0.907	0.530	0.039	0.283	0.537	−0.004	0.757	0.457	0.008	0.464
Time in bouts of 45 min	0.469	0.000	0.775	0.534	0.001	0.199	0.537	0.000	0.683	0.459	0.000	0.389
Model 2												
Sedentary time	0.465	0.000	0.471	0.544	0.000	0.604	0.538	0.000	0.732	0.456	0.000	0.427
Bouts of 10 min	0.462	0.001	0.676	0.543	−0.001	0.635	0.537	0.000	0.954	0.451	0.000	0.977
Time in bouts of 10 min	0.461	0.000	0.856	0.542	0.000	0.806	0.537	0.000	0.903	0.452	0.000	0.768
Bouts of 20 min	0.461	−0.001	0.819	0.542	0.000	0.980	0.538	0.000	0.882	0.451	0.000	0.860
Time in bouts of 20 min	0.461	0.000	0.800	0.542	0.000	0.822	0.538	0.000	0.808	0.452	0.000	0.679
Bouts of 30 min	0.461	0.001	0.918	0.543	0.006	0.752	0.537	0.000	0.957	0.452	0.002	0.697
Time in bouts of 30 min	0.461	0.000	0.924	0.544	0.000	0.581	0.538	0.000	0.849	0.454	0.000	0.509
Bouts of 45 min	0.461	−0.003	0.896	0.544	0.021	0.578	0.538	−0.004	0.714	0.457	0.010	0.374
Time in bouts of 45 min	0.462	0.000	0.762	0.546	0.000	0.466	0.539	0.000	0.631	0.459	0.000	0.301

β, standardized coefficient; DSB, daily sedentary behaviors; SB, sedentary behaviors; and WSB, weekend sedentary behaviors. Model 1: Analyses were controlled by age, educational center, mother’s education level, and metabolic syndrome score at baseline. Model 2: Model 1 plus moderate to vigorous physical activity time at baseline. Statistically significant values are highlighted in bold.

**Table 4:** Association between changes in sedentary behaviors and sedentary patterns with metabolic syndrome at 2-year follow-ups.

Change in sedentary behaviors												
	Children (*n* = 76)						Adolescents (*n* = 186)					
	Male (*n* = 42)			Female (*n* = 34)			Male (*n* = 92)			Female (*n* = 94)		
	Adjusted R^2^	β	*p*	Adjusted R^2^	β	*p*	Adjusted R^2^	β	*p*	Adjusted R^2^	β	*p*
Model 1												
Total DSB	0.273	0.000	0.858	0.220	0.000	0.665	0.553	0.000	0.190	0.440	0.000	0.847
Screen DSB	0.274	0.000	0.816	0.211	0.000	0.977	0.553	0.000	0.173	0.477	0.001	**0.023**
Educative DSB	0.272	0.000	0.971	0.222	0.000	0.626	0.548	0.000	0.313	0.473	−0.001	**0.033**
Social DSB	0.272	0.000	0.966	0.212	0.000	0.908	0.553	−0.001	0.176	0.440	0.000	0.771
Other DSB	0.272	0.000	0.917	0.245	0.005	0.396	0.543	0.000	0.639	0.471	0.002	**0.037**
Total WSB	0.278	0.000	0.645	0.212	0.000	0.871	0.543	0.000	0.614	0.448	0.000	0.282
Screen WSB	0.274	0.000	0.801	0.240	0.000	0.429	0.548	0.000	0.333	0.460	0.001	0.097
Educative WSB	0.277	0.000	0.688	0.235	−0.001	0.474	0.546	0.000	0.395	0.441	0.000	0.655
Social WSB	0.289	0.000	0.460	0.287	0.001	0.197	0.545	0.000	0.476	0.440	0.000	0.881
Other WSB	0.342	−0.002	0.122	0.252	0.001	0.345	0.553	−0.001	0.189	0.443	0.001	0.470
Mean SB	0.275	0.000	0.742	0.218	0.000	0.695	0.546	0.000	0.438	0.442	0.000	0.534
Model 2												
Total DSB	0.242	0.000	0.870	0.230	0.000	0.846	0.546	0.000	0.199	0.442	0.000	0.940
Screen DSB	0.243	0.000	0.851	0.245	0.001	0.560	0.547	0.000	0.181	0.475	0.001	**0.033**
Educative DSB	0.241	0.000	0.960	0.228	0.000	0.947	0.542	0.000	0.319	0.474	−0.001	**0.037**
Social DSB	0.241	0.000	0.982	0.235	−0.001	0.718	0.547	−0.001	0.182	0.444	0.000	0.586
Other DSB	0.242	0.000	0.943	0.254	0.004	0.466	0.537	0.000	0.637	0.475	0.002	**0.035**
Total WSB	0.248	0.000	0.661	0.228	0.000	0.982	0.538	0.000	0.584	0.449	0.000	0.315
Screen WSB	0.243	0.000	0.815	0.246	0.000	0.548	0.541	0.000	0.347	0.457	0.000	0.153
Educative WSB	0.246	0.000	0.717	0.267	−0.001	0.372	0.540	0.000	0.387	0.442	0.000	0.792
Social WSB	0.260	0.000	0.461	0.275	0.001	0.323	0.539	0.000	0.489	0.442	0.000	0.832
Other WSB	0.314	−0.002	0.131	0.276	0.001	0.319	0.547	−0.001	0.193	0.446	0.001	0.442
Mean SB	0.245	0.000	0.758	0.229	0.000	0.880	0.539	0.000	0.457	0.443	0.000	0.699
Sedentary patterns												
	Children (*n* = 175)						Adolescents (*n* = 188)					
	Male (*n* = 93)			Female (*n* = 82)			Male (*n* = 93)			Female (*n* = 95)		
	Adjusted R^2^	β	*p*	Adjusted R^2^	β	*p*	Adjusted R^2^	β	*p*	Adjusted R^2^	β	*p*
Model 1												
Sedentary time	0.469	0.000	0.695	0.523	0.000	0.706	0.537	0.000	0.782	0.461	0.000	0.290
Bouts of 10 min	0.468	0.000	0.876	0.534	0.003	0.202	0.536	0.000	0.925	0.456	−0.001	0.534
Time in bouts of 10 min	0.468	0.000	0.930	0.532	0.000	0.238	0.536	0.000	0.855	0.457	0.000	0.469
Bouts of 20 min	0.469	0.001	0.769	0.529	0.005	0.327	0.536	0.000	0.986	0.455	−0.001	0.639
Time in bouts of 20 min	0.468	0.000	0.867	0.527	0.000	0.387	0.536	0.000	0.821	0.456	0.000	0.544
Bouts of 30 min	0.470	−0.005	0.600	0.525	0.006	0.503	0.537	−0.002	0.763	0.454	−0.001	0.806
Time in bouts of 30 min	0.469	0.000	0.785	0.525	0.000	0.524	0.536	0.000	0.861	0.455	0.000	0.620
Bouts of 45 min	0.468	0.001	0.952	0.522	0.003	0.867	0.536	0.000	0.956	0.454	−0.002	0.819
Time in bouts of 45 min	0.468	0.000	0.871	0.522	0.000	0.864	0.537	0.000	0.797	0.454	0.000	0.708
Model 2												
Sedentary time	0.462	0.000	0.742	0.515	0.000	0.754	0.531	0.000	0.728	0.459	0.000	0.506
Bouts of 10 min	0.462	0.000	0.844	0.526	0.003	0.213	0.530	0.000	0.957	0.456	0.000	0.732
Time in bouts of 10 min	0.461	0.000	0.908	0.525	0.000	0.247	0.531	0.000	0.833	0.457	0.000	0.722
Bouts of 20 min	0.462	0.001	0.756	0.522	0.005	0.331	0.530	0.000	0.998	0.456	0.000	0.909
Time in bouts of 20 min	0.462	0.000	0.857	0.520	0.000	0.387	0.531	0.000	0.807	0.456	0.000	0.835
Bouts of 30 min	0.463	−0.005	0.599	0.518	0.007	0.497	0.531	−0.001	0.790	0.456	0.001	0.910
Time in bouts of 30 min	0.462	0.000	0.785	0.518	0.000	0.514	0.531	0.000	0.845	0.456	0.000	0.893
Bouts of 45 min	0.461	0.001	0.944	0.515	0.004	0.835	0.530	0.000	0.984	0.456	0.001	0.913
Time in bouts of 45 min	0.461	0.000	0.865	0.515	0.000	0.834	0.531	0.000	0.785	0.456	0.000	0.935

β, standardized coefficient; DSB, daily sedentary behaviors; SB, sedentary behaviors; and WSB, weekend sedentary behaviors. Model 1: Analyses were controlled by age, educational center, mother’s education level, and metabolic syndrome score at baseline. Model 2: Model 1 plus changes in moderate to vigorous physical activity time. Statistically significant values are highlighted in bold.

## Data Availability

Data available on request due to restrictions.

## Supplementary Materials

The following supporting information can be downloaded at: https://www.mdpi.com/article/10.3390/healthcare13192544/s1. Table S1: Changes in characteristics of the study sample by age and sex (Follow-up—Baseline). Table S2: Association between changes in sedentary behaviors and sedentary patterns with changes in metabolic syndrome. Table S3: Definitions and abbreviations of study variables.
