# Supplementary material for: Associations Between Sedentary Behaviors and Sedentary Patterns with Metabolic Syndrome in Children and Adolescents: The UP&DOWN Longitudinal Study
Source: Healthcare (Basel). 2025 Oct 9;13(19):2544. doi: 10.3390/healthcare13192544 (PMC12524390; doi:10.3390/healthcare13192544)
Supplement: Supplementary file 1 [file healthcare-13-02544-s001.zip › Table S1.pdf]

Table S1. Changes in characteristics of the study sample by age and sex (Follow-up - Baseline).

	**Male children**	**Female children**	**P value**	**Male adolescents**	**Female adolescents**	**P value**
Sedentary behaviors	(*n* = 42)	(*n* = 34)		(*n* = 92)	(*n* = 94)	
Total DSB (min/day)	23.98 (173.61)	-17.21 (141.80)	0.269	45.99 (153.01)	62.95 (134.53)	0.423
Screen DSB (min/day)	3.66 (143.75)	19.20 (78.74)	0.574	-12.45 (136.05)	-6.46 (131.20)	0.760
Educative DSB (min/day)	12.45 (150.11)	-19.69 (140.90)	0.343	27.30 (164.98)	49.59 (181.67)	0.382
Social DSB (min/day)	-2.42 (62.98)	-12.13 (73.09)	0.536	31.40 (86.24)	29.22 (103.66)	0.877
Other DSB (min/day)	10.29 (63.53)	-4.59 (26.56)	0.205	-0.26 (47.65)	-9.40 (58.49)	0.245
Total WSB (min/day)	22.80 (245.15)	22.47 (240.21)	0.995	60.82 (204.16)	41.22 (201.90)	0.511
Screen WSB (min/day)	22.32 (206.77)	9.80 (217.50)	0.798	-7.92 (210.10)	-55.27 (159.77)	0.085
Educative WSB (min/day)	24.02 (111.98)	2.84 (102.55)	0.397	45.73 (186.61)	79.75 (196.72)	0.228
Social WSB (min/day)	-12.45 (124.35)	15.44 (153.67)	0.385	30.94 (108.42)	37.62 (132.18)	0.707
Other WSB (min/day)	-11.10 (66.01)	-5.61 (88.62)	0.758	-7.93 (58.27)	-20.87 (68.64)	0.168
Mean SB (min/day)	23.65 (156.40)	-5.87 (141.84)	0.397	50.23 (137.72)	56.74 (126.11)	0.737
Sedentary pattern	(*n* = 93)	(*n* = 82)		(*n* = 93)	(*n* = 95)	
Sedentary (min/day)	37.77 (64.76)	36.13 (73.86)	0.876	188.70 (447.42)	189.70 (380.32)	0.987
Bouts 10 min (number/day)	2.15 (3.03)	2.09 (3.17)	0.899	2.00 (5.31)	2.55 (4.34)	0.445
Time in Bouts 10 min (min/day)	32.61 (67.87)	42.40 (68.97)	0.346	192.02 (412.05)	207.96 (350.69)	0.775
Average in Bouts 10 min (min/bouts)	33.90 (69.58)	43.61 (71.07)	0.363	113.25 (231.77)	138.92 (205.27)	0.422
Bouts 20 min (number/day)	0.37 (1.44)	0.72 (1.50)	0.117	1.19 (2.84)	1.54 (2.71)	0.381
Time in Bouts 20 min (min/day)	8.62 (48.93)	22.73 (51.49)	0.065	180.23 (394.53)	193.80 (338.97)	0.800
Average in Bouts 20 min (min/bouts)	9.20 (50.19)	23.43 (53.37)	0.071	110.56 (228.50)	133.14 (209.68)	0.481
Bouts 30 min (number/day)	0.05 (0.76)	0.29 (0.80)	**0.046**	0.58 (1.66)	1.06 (1.82)	0.060
Time in Bouts 30 min (min/day)	1.33 (34.24)	12.02 (35.36)	**0.044**	165.76 (381.79)	182.09 (331.21)	0.754
Average in Bouts 30 min (min/bouts)	1.64 (35.12)	12.43 (36.39)	**0.048**	100.83 (222.65)	125.82 (209.08)	0.428
Bouts 45 min (number/day)	-0.01 (0.35)	0.09 (0.39)	0.073	0.37 (1.08)	0.64 (1.18)	0.114
Time in Bouts 45 min (min/day)	-0.82 (20.92)	4.91 (21.87)	0.079	158.42 (372.45)	166.70 (319.18)	0.870
Average in Bouts 45 min (min/bouts)	-0.70 (21.34)	5.08 (22.30)	0.082	96.74 (218.65)	114.88 (204.61)	0.557
Physical activity						
MVPA (min/day)	-9.17 (20.35)	-0.67 (15.95)	**0.003**	-5.24 (23.46)	-2.92 (17.43)	0.443
Tanner stage	0.72 (0.63)	0.72 (0.76)	0.993	0.91 (0.90)	0.57 (0.72)	**0.004**
Age (years)	2.03 (0.08)	2.05 (0.08)	0.131	2.00 (0.11)	2.00 (0.07)	0.708
Systolic blood pressure (mmHg)	3.68 (10.37)	5.61 (9.93)	0.211	3.62 (11.40)	-3.02 (11.59)	**<0.001**
Triglycerides (mg/dL)	1.97 (25.45)	0.79 (23.76)	0.754	14.46 (26.07)	9.58 (24.25)	0.185
HDL Cholesterol (mg/dL)	0.04 (19.27)	-0.22 (21.98)	0.933	3.57 (14.25)	9.61 (15.52)	**0.006**
Glucose (mg/dL)	10.61 (25.31)	11.63 (22.79)	0.781	7.03 (17.00)	6.93 (16.04)	0.965
Body composition						
Weight (kg)	8.00 (3.17)	8.65 (4.60)	0.274	9.04 (5.24)	4.40 (5.33)	**<0.001**
Height (cm)	11.43 (1.94)	12.95 (2.68)	**<0.001**	10.12 (6.61)	4.35 (3.55)	**<0.001**
Body mass index (kg/m^2)	1.18 (1.23)	0.99 (1.55)	0.346	0.84 (1.50)	0.65 (1.72)	0.414
Waist circumference (cm)	4.28 (3.49)	3.02 (3.80)	**0.024**	3.11 (3.68)	1.31 (3.40)	**0.001**

HDL, high density lipoprotein; MVPA, moderate to vigorous physical activity; DSB, daily sedentary behaviors; WSB, weekend sedentary behaviors; SB, sedentary behaviors.

Values are presented as mean (standard deviation). Statistically significant differences between sex in variables are highlighted in bold.
