# Supplementary material for: Associations Between Sedentary Behaviors and Sedentary Patterns with Metabolic Syndrome in Children and Adolescents: The UP&DOWN Longitudinal Study
Source: Healthcare (Basel). 2025 Oct 9;13(19):2544. doi: 10.3390/healthcare13192544 (PMC12524390; doi:10.3390/healthcare13192544)
Supplement: Supplementary file 1 [file healthcare-13-02544-s001.zip › Table S2.pdf]

**Table S2.** Association between changes in sedentary behaviors and sedentary patterns with changes in metabolic syndrome.

| Change in sedentary behaviors | | | | | | | | | | | | |
|---|---|---|---|---|---|---|---|---|---|---|---|---|
| | **Children** (*n* = 76) | | | | | | **Adolescents** (*n* = 186) | | | | | |
| | **Male** (*n* = 42) | | | **Female** (*n* = 34) | | | **Male** (*n* =92) | | | **Female** (*n* = 94) | | |
| | Adjusted $R^2$ | β | p | Adjusted $R^2$ | β | p | Adjusted $R^2$ | β | p | Adjusted $R^2$ | β | p |
| *Model 1* | | | | | | | | | | | | |
| Total DSB | 0.088 | 0.000 | 0.872 | -0.034 | 0.000 | 0.707 | 0.151 | 0.000 | 0.393 | 0.045 | 0.000 | 0.852 |
| Screen DSB | 0.104 | 0.000 | 0.496 | -0.016 | -0.001 | 0.508 | 0.163 | 0.000 | 0.183 | 0.121 | 0.001 | **0.013** |
| Educative DSB | 0.097 | 0.000 | 0.599 | -0.021 | -0.001 | 0.551 | 0.146 | 0.000 | 0.581 | 0.114 | -0.001 | **0.018** |
| Social DSB | 0.093 | -0.001 | 0.681 | -0.013 | 0.001 | 0.478 | 0.169 | -0.001 | 0.128 | 0.045 | 0.000 | 0.871 |
| Other DSB | 0.101 | -0.001 | 0.536 | 0.037 | 0.007 | 0.240 | 0.142 | 0.000 | 0.974 | 0.096 | 0.002 | **0.042** |
| Total WSB | 0.088 | 0.000 | 0.868 | -0.038 | 0.000 | 0.785 | 0.147 | 0.000 | 0.538 | 0.055 | 0.000 | 0.374 |
| Screen WSB | 0.088 | 0.000 | 0.904 | 0.102 | -0.001 | 0.106 | 0.153 | 0.000 | 0.337 | 0.077 | 0.001 | 0.109 |
| Educative WSB | 0.105 | 0.000 | 0.486 | -0.002 | -0.001 | 0.407 | 0.155 | 0.000 | 0.292 | 0.046 | 0.000 | 0.726 |
| Social WSB | 0.094 | 0.000 | 0.667 | 0.134 | 0.001 | 0.072 | 0.146 | 0.000 | 0.543 | 0.048 | 0.000 | 0.629 |
| Other WSB | 0.145 | -0.002 | 0.203 | 0.038 | 0.002 | 0.236 | 0.159 | -0.001 | 0.226 | 0.052 | 0.001 | 0.438 |
| Mean SB | 0.087 | 0.000 | 0.951 | -0.033 | 0.000 | 0.691 | 0.144 | 0.000 | 0.704 | 0.048 | 0.000 | 0.592 |
| *Model 2* | | | | | | | | | | | | |
| Total DSB | 0.051 | 0.000 | 0.866 | -0.044 | 0.000 | 0.552 | 0.139 | 0.000 | 0.418 | 0.047 | 0.000 | 0.948 |
| Screen DSB | 0.067 | 0.000 | 0.521 | -0.054 | -0.001 | 0.663 | 0.151 | 0.000 | 0.198 | 0.116 | 0.001 | **0.019** |
| Educative DSB | 0.060 | 0.000 | 0.615 | 0.010 | -0.001 | 0.268 | 0.135 | 0.000 | 0.589 | 0.114 | -0.001 | **0.020** |
| Social DSB | 0.056 | -0.001 | 0.699 | -0.041 | 0.001 | 0.528 | 0.158 | -0.001 | 0.134 | 0.049 | 0.000 | 0.692 |
| Other DSB | 0.064 | -0.001 | 0.559 | 0.010 | 0.006 | 0.267 | 0.131 | 0.000 | 0.983 | 0.100 | 0.002 | **0.040** |
| Total WSB | 0.051 | 0.000 | 0.881 | -0.056 | 0.000 | 0.692 | 0.137 | 0.000 | 0.496 | 0.055 | 0.000 | 0.411 |
| Screen WSB | 0.051 | 0.000 | 0.895 | 0.080 | -0.001 | 0.118 | 0.142 | 0.000 | 0.361 | 0.071 | 0.000 | 0.166 |
| Educative WSB | 0.068 | 0.000 | 0.510 | -0.010 | -0.001 | 0.344 | 0.145 | 0.000 | 0.281 | 0.047 | 0.000 | 0.860 |
| Social WSB | 0.058 | 0.000 | 0.663 | 0.093 | 0.001 | 0.101 | 0.136 | 0.000 | 0.564 | 0.050 | 0.000 | 0.587 |
| Other WSB | 0.110 | -0.002 | 0.216 | 0.026 | 0.002 | 0.220 | 0.149 | -0.001 | 0.230 | 0.055 | 0.001 | 0.413 |
| Mean SB | 0.050 | 0.000 | 0.941 | -0.042 | 0.000 | 0.537 | 0.133 | 0.000 | 0.748 | 0.048 | 0.000 | 0.753 |

| Sedentary patterns | | | | | | | | | | | | |
|---|---|---|---|---|---|---|---|---|---|---|---|---|
| | **Children** (*n* = 175) | | | | | | **Adolescents** (*n* = 188) | | | | | |
| | **Male** (*n* = 93) | | | **Female** (*n* = 82) | | | **Male** (*n* = 93) | | | **Female** (*n* = 95) | | |
| | Adjusted $R^2$ | β | p | Adjusted $R^2$ | β | p | Adjusted $R^2$ | β | p | Adjusted $R^2$ | β | p |
| *Model 1* | | | | | | | | | | | | |
| Sedentary time | 0.159 | 0.000 | 0.899 | 0.016 | 0.000 | 0.844 | 0.154 | 0.000 | 0.935 | 0.049 | 0.000 | 0.611 |
| Bouts 10 min | 0.164 | 0.001 | 0.490 | 0.031 | 0.003 | 0.310 | 0.157 | -0.001 | 0.592 | 0.046 | 0.000 | 0.900 |
| Time in Bouts 10 min | 0.163 | 0.000 | 0.533 | 0.023 | 0.000 | 0.480 | 0.154 | 0.000 | 0.957 | 0.046 | 0.000 | 0.904 |
| Average in Bouts 10 min | 0.161 | 0.000 | 0.638 | 0.026 | 0.001 | 0.398 | 0.154 | 0.000 | 0.786 | 0.058 | 0.000 | 0.324 |
| Bouts 20 min | 0.167 | 0.004 | 0.380 | 0.017 | 0.002 | 0.707 | 0.159 | -0.002 | 0.486 | 0.046 | 0.000 | 0.953 |
| Time in Bouts 20 min | 0.163 | 0.000 | 0.500 | 0.016 | 0.000 | 0.823 | 0.154 | 0.000 | 0.980 | 0.046 | 0.000 | 0.835 |

| | | | | | | | | | | | | |
|---|---|---|---|---|---|---|---|---|---|---|---|---|
| Average in Bouts 20 min | 0.162 | 0.001 | 0.543 | 0.017 | 0.000 | 0.760 | 0.154 | 0.000 | 0.883 | 0.050 | 0.000 | 0.550 |
| Bouts 30 min | 0.158 | 0.001 | 0.932 | 0.015 | -0.001 | 0.910 | 0.162 | -0.005 | 0.386 | 0.046 | 0.001 | 0.824 |
| Time in Bouts 30 min | 0.159 | 0.000 | 0.841 | 0.015 | 0.000 | 0.928 | 0.154 | 0.000 | 0.908 | 0.046 | 0.000 | 0.902 |
| Average in Bouts 30 min | 0.159 | 0.000 | 0.835 | 0.015 | 0.000 | 0.932 | 0.154 | 0.000 | 0.980 | 0.050 | 0.000 | 0.574 |
| Bouts 45 min | 0.160 | 0.007 | 0.727 | 0.015 | -0.003 | 0.874 | 0.157 | -0.005 | 0.584 | 0.046 | 0.001 | 0.906 |
| Time in Bouts 45 min | 0.160 | 0.000 | 0.690 | 0.015 | 0.000 | 0.884 | 0.154 | 0.000 | 0.772 | 0.046 | 0.000 | 0.872 |
| Average in Bouts 45 min | 0.161 | 0.001 | 0.605 | 0.015 | 0.000 | 0.954 | 0.154 | 0.000 | 0.812 | 0.047 | 0.000 | 0.790 |
| *Model 2* | | | | | | | | | | | | |
| Sedentary time | 0.147 | 0.000 | 0.928 | 0.000 | 0.000 | 0.874 | 0.144 | 0.000 | 0.866 | 0.047 | 0.000 | 0.903 |
| Bouts 10 min | 0.153 | 0.002 | 0.473 | 0.017 | 0.003 | 0.300 | 0.146 | -0.001 | 0.627 | 0.049 | 0.001 | 0.691 |
| Time in Bouts 10 min | 0.152 | 0.000 | 0.522 | 0.008 | 0.000 | 0.475 | 0.144 | 0.000 | 0.986 | 0.047 | 0.000 | 0.810 |
| Average in Bouts 10 min | 0.150 | 0.000 | 0.641 | 0.011 | 0.001 | 0.405 | 0.145 | 0.000 | 0.778 | 0.064 | 0.001 | 0.247 |
| Bouts 20 min | 0.156 | 0.004 | 0.376 | 0.002 | 0.002 | 0.706 | 0.149 | -0.002 | 0.505 | 0.048 | 0.001 | 0.779 |
| Time in Bouts 20 min | 0.153 | 0.000 | 0.497 | 0.001 | 0.000 | 0.825 | 0.144 | 0.000 | 0.960 | 0.047 | 0.000 | 0.863 |
| Average in Bouts 20 min | 0.151 | 0.001 | 0.547 | 0.001 | 0.000 | 0.768 | 0.144 | 0.000 | 0.883 | 0.056 | 0.000 | 0.383 |
| Bouts 30 min | 0.147 | 0.001 | 0.933 | 0.000 | -0.001 | 0.907 | 0.152 | -0.004 | 0.412 | 0.051 | 0.003 | 0.570 |
| Time in Bouts 30 min | 0.148 | 0.000 | 0.842 | 0.000 | 0.000 | 0.922 | 0.144 | 0.000 | 0.887 | 0.047 | 0.000 | 0.825 |
| Average in Bouts 30 min | 0.148 | 0.000 | 0.840 | 0.000 | 0.000 | 0.942 | 0.144 | 0.000 | 0.990 | 0.055 | 0.001 | 0.406 |
| Bouts 45 min | 0.149 | 0.007 | 0.724 | 0.000 | -0.004 | 0.857 | 0.147 | -0.005 | 0.615 | 0.049 | 0.004 | 0.661 |
| Time in Bouts 45 min | 0.149 | 0.000 | 0.689 | 0.000 | 0.000 | 0.868 | 0.145 | 0.000 | 0.756 | 0.047 | 0.000 | 0.913 |
| Average in Bouts 45 min | 0.150 | 0.001 | 0.606 | 0.000 | 0.000 | 0.934 | 0.144 | 0.000 | 0.798 | 0.050 | 0.000 | 0.623 |

DSB, daily sedentary behaviors; WSB, weekend sedentary behaviors; SB, sedentary behaviors; β, standardized coefficient. Model 1: Analyses were controlled by age, educational centre and mother´s education level at baseline. Model 2: model 1 plus changes in moderate to vigorous physical activity. Statistically significant values are highlighted in bold.
