# Supplementary material for: Associations Between Sedentary Behaviors and Sedentary Patterns with Metabolic Syndrome in Children and Adolescents: The UP&DOWN Longitudinal Study
Source: Healthcare (Basel). 2025 Oct 9;13(19):2544. doi: 10.3390/healthcare13192544 (PMC12524390; doi:10.3390/healthcare13192544)
Supplement: Supplementary file 1 [file healthcare-13-02544-s001.zip › Table S3.pdf]

Table S3: Definitions and abbreviations of study variables.

Variable	Abbreviation in tables	Definition
Total daily sedentary behavior	Total DSB (min/day)	Total minutes per day spent in sedentary behaviors on weekdays.
Screen daily sedentary behavior	Screen DSB (min/day)	Minutes per day spent watching TV, playing video games, or using computers/smartphones for leisure during weekdays.
Educative daily sedentary behavior	Educative DSB (min/day)	Minutes per day spent in sedentary educational activities (e.g., studying, doing homework, reading) on weekdays.
Social daily sedentary behavior	Social DSB (min/day)	Minutes per day spent in sedentary social activities (e.g., talking) on weekdays.
Other daily sedentary behavior	Other DSB (min/day)	Minutes per day spent in other sedentary behaviors not included in the categories above, on weekdays.
Total weekend sedentary behavior	Total WSB (min/day)	Total minutes per day spent in sedentary behaviors on weekends.
Screen weekend sedentary behavior	Screen WSB (min/day)	Minutes per day spent watching TV, playing video games, or using computers/smartphones for leisure during weekends.
Educative weekend sedentary behavior	Educative WSB (min/day)	Minutes per day spent in sedentary educational activities during weekends.
Social weekend sedentary behavior	Social WSB (min/day)	Minutes per day spent in sedentary social activities during weekends.
Other weekend sedentary behavior	Other WSB (min/day)	Minutes per day spent in other sedentary behaviors on weekends.
Mean sedentary behavior	Mean SB (min/day)	Average sedentary behavior minutes combining weekdays and weekends.
Accelerometer wear time	Accelerometer wear time (h/day)	Average daily time (hours) participants wore the accelerometer.
Sedentary time	Sedentary time (min/day)	Average minutes per day spent in sedentary time as measured by accelerometer.
Bouts ≥10 min	Bouts 10 min (number/day)	Average number of sedentary bouts lasting at least 10 consecutive minutes per day.
Time in bouts ≥10 min	Time in Bouts 10 min (min/day)	Minutes per day accumulated in sedentary bouts ≥10 minutes.
Moderate-to-vigorous physical activity	MVPA (min/day)	Minutes per day accumulated in moderate-to-vigorous physical activity measured by accelerometer.
Tanner stage	Tanner stage	Pubertal maturation stage self-reported according to Tanner's scale.
Age	Age (years)	Chronological age in years.
Systolic blood pressure	Systolic blood pressure (mmHg)	Average systolic blood pressure in mmHg.
Triglycerides	Triglycerides (mg/dL)	Serum triglycerides concentration (mg/dL).
HDL cholesterol	HDL cholesterol (mg/dL)	Serum high-density lipoprotein cholesterol concentration (mg/dL).
Glucose	Glucose (mg/dL)	Serum glucose concentration (mg/dL).
Weight	Weight (kg)	Body weight in kilograms.
Height	Height (cm)	Height in centimeters.
Body mass index	BMI (kg/m^2)	Body mass index, calculated as weight (kg) / height (m^2).
